# Using Kalirin conditional knockout mice to distinguish its role in dopamine receptor mediated behaviors

**DOI:** 10.1186/s12868-017-0363-2

**Published:** 2017-05-23

**Authors:** Taylor P. LaRese, Yan Yan, Betty A. Eipper, Richard E. Mains

**Affiliations:** 1 Department of Neuroscience, University of Connecticut Health Center, Farmington, CT 06030-3401 USA; 2 Departments of Neuroscience and Molecular Biology and Biophysics, University of Connecticut Health Center, Farmington, CT 06030-3401 USA

**Keywords:** Cre recombinase, GDP/GTP exchange factor, GEF, Trio, Anxiety, Locomotor sensitization

## Abstract

**Background:**

Mice lacking Kalirin-7 (Kal7^KO^), a Rho GDP/GTP exchange factor, self-administer cocaine at a higher rate than wildtype mice, and show an exaggerated locomotor response to experimenter-administered cocaine. Kal7, which localizes to post-synaptic densities at glutamatergic synapses, interacts directly with the GluN2B subunit of the *N*-methyl-d-aspartate (NMDA; GluN) receptor. Consistent with these observations, Kal7 plays an essential role in NMDA receptor dependent long term potentiation and depression, and glutamatergic transmission plays a key role in the response to chronic cocaine. A number of genetic studies have implicated altered Kalirin expression in schizophrenia and other disorders such as Alzheimer’s Disease.

**Results:**

A comparison of the effects of experimenter-administered cocaine on mice lacking all Kalirin isoforms to its effects on mice lacking only Kalirin-7 identified Kal7 as the key isoform whose deletion produces exaggerated locomotor responses to cocaine. Pretreatment of Kal7^KO^ mice with a low dose of ifenprodil, a selective GluN2B antagonist, eliminated their enhanced locomotor response to cocaine, revealing an important role for GluN2B in this behavior. Selective knockout of Kalirin in dopamine transporter expressing neurons produced a transient enhancement of cocaine-induced locomotion, while knockout of Kalirin in Drd1a- or Drd2-dopamine receptor expressing neurons was without effect. As observed in Kalirin global knockout mice, eliminating Kalirin expression in Drd2-expressing neurons increased exploratory behavior in the elevated zero maze, an effect eliminated by pretreatment with ifenprodil.

**Conclusions:**

The cocaine-sensitive neuronal pathways which are most sensitive to altered Kalirin function may be the pathways most dependent on GluN2B and Drd2.

**Electronic supplementary material:**

The online version of this article (doi:10.1186/s12868-017-0363-2) contains supplementary material, which is available to authorized users.

## Background

**Fig. 1:**
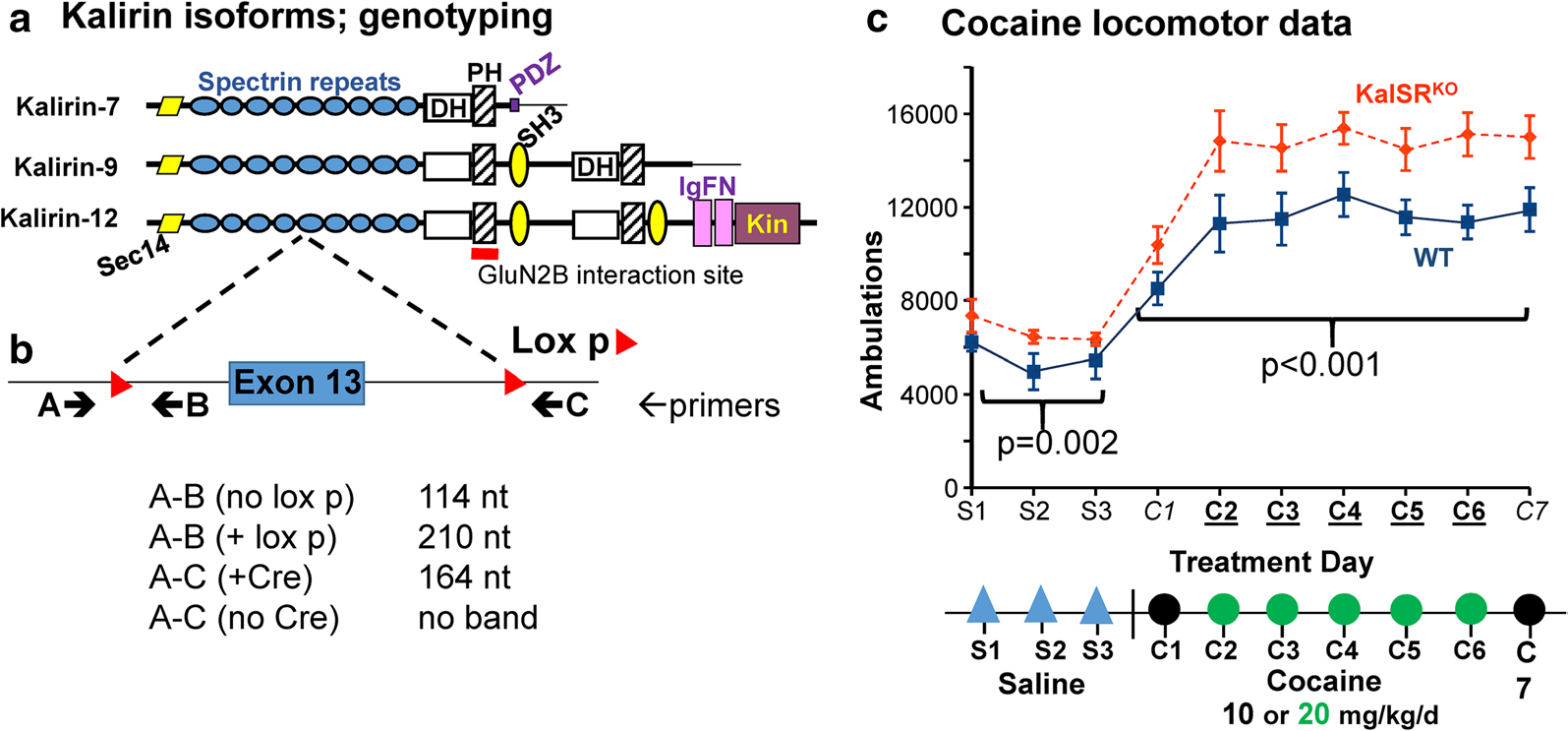
KalSR^KO^ mice exhibit enhanced locomotor sensitization to experimenter-administered cocaine. **a** Diagram showing the major Kalirin protein isoforms in adult rodents; the region of Kalirin shown to bind to the juxtamembrane region of GluN2B is shown. Sec14, phospholipid binding domain; DH, catalytically active Dbl homology GDP/GTP exchange factor domain; PH, pleckstrin homology domain; SH3, protein–protein interaction Src Homology 3 domain; Ig, immunoglobulin-like domain; FN, fibronectin-like domain; Kin, Ser/Thr kinase domain. **b** Diagram showing the genomic region surrounding *Kalrn* Exon 13 and the placement of forward (→) and reverse (←) primers used for genotyping WT, KalSR^CKO^ and KalSR^KO^ mice. **c** Adult male mice (N = 7 each, WT and KalSR^KO^) were studied every day for 2 weeks. Mice were initially handled for 3 days, injected with saline for 3 days (S1–S3), and then injected with cocaine i.p. [10 mg/kg, days 1 and 7; 20 mg/kg days 2–6 [9, 74]; C1–C7]; locomotor activity was recorded for 45 min after each injection. Mice were then maintained without injections for 12 days and tested again with 10 mg/kg cocaine (not shown). Males only, N = 7 each genotype. Statistics, SigmaPlot using repeated measures ANOVA, p < 0.001)

Cocaine has an addictive allure matched only by the complexity of its neurochemical effects [1–4]. At its simplest, cocaine blocks the dopamine reuptake transporter (DAT); at its more complex and realistic, cocaine attacks many neurotransmitter systems in the brain [1–5]. Mammalian Kalirin and Trio form a two member family of multifunctional proteins with both scaffolding domains and enzymatic domains (Fig. 1a); lower organisms have a single member of this family (e.g. *Unc-73* and *dTrio*) [6]. Kalirin deficiency in the Kalirin-7 knockout mouse (Kal7^KO^) leads to greatly exaggerated cocaine self-administration and an accentuated locomotor response to cocaine administration [7–9]. Kalirin transcript levels are increased by chronic cocaine and remain elevated during withdrawal [10]. The first pleckstrin homology (PH1) domain of Kalirin interacts directly with the NMDA receptor subunit GluN2B (formerly called NR2B; the product of the Grin2b gene), and Kalirin overexpression stimulates dendritic spine growth in many neuronal types; conversely, Kalirin loss blunts dendritic spine growth [7, 11–15]. Thus, it was important to investigate which neurotransmitter systems were most relevant to the self-administration and locomotor responses to cocaine which are enhanced by Kalirin deficiency. In addition, Kalirin deficiencies have been implicated in a number of inherited disorders such as schizophrenia and Alzheimer’s Disease [16–20].

Electrophysiological studies demonstrated that Kal7^KO^ neurons showed markedly suppressed long-term potentiation (LTP) to theta burst stimulation (a physiologically relevant stimulation pattern) in CA1 hippocampal slices, with normal neuronal electrical membrane properties [12]. Subsequent studies confirmed the dramatic loss of long-term potentiation, and added the observation that long-term depression was abolished in Kal7^KO^ mice [11]. Using stimulation protocols that can also activate GluN receptor-independent LTP [21, 22], these data were questioned; however, detailed analyses demonstrated that indeed GluN receptor-independent LTP was normal in Kal7^KO^ mice while GluN receptor-dependent LTP was dramatically impaired [11]. These findings have been confirmed in studies of spinal cord pain perception, which demonstrated that Kal7^KO^ mice lose nociceptor-dependent LTP, with corresponding blunting of nocifensive behavior (avoiding pain), compared to wildtype mice [23]. Similarly, mice injected in the spinal cord with siRNA targeting Kalirin had depressed allodynia [24]. Extending these findings, intracellular injection of a Kal7-specific interfering peptide (for which there is no Trio counterpart) totally blocked spinal pain LTP [23], and pharmacologic blockade of Kalirin and Trio guanine nucleotide exchange factor 1 (GEF1) eliminated both long-term potentiation and long-term depression in hippocampal slices [11]. Together, the data from at least four studies indicate that Kal7 is crucial for several types of LTP and LTD.

Chronic exposure to cocaine is known to produce multiple variations in glutamatergic signaling [25, 26]. In wildtype mice, expression of transcripts encoding GluN2B and Kal7 increases during both cocaine self-administration and chronic cocaine administration [8, 10]. Kal7 and GluN2B are localized to the post-synaptic densities of glutamatergic synapses [13, 27], focusing our attention on synaptic transmission. Importantly, our earlier electrophysiological and biochemical studies revealed an essential role for Kal7 in responses that involve GluN receptors which contain the GluN2B subunit [11, 28]. Administration of a low dose of ifenprodil (2 mg/kg i.p.), a GluN2B blocker, 10 min before injection of saline or cocaine abrogated the differences in cocaine conditioned place preference between WT and Kal7^KO^ mice; ifenprodil administration also eliminated any genotypic difference in passive avoidance fear conditioning [28]. This dose of ifenprodil was without effect on locomotor activity or the increase in locomotion observed in response to a single injection of cocaine [28].

The current investigation queried the roles of dopaminergic and glutamatergic pathways in the behavioral responses to cocaine in mice with deletions of only Kal7 (Kal7^KO^) or the totality of Kalirin-related transcripts (Kalirin spectrin-repeat knockout; KalSR^KO^), either in the whole animal or in subsets of neurons which could be informative about Kalirin function. We employed and verified Cre-recombinase mouse lines capable of eliminating Kalirin expression in cells expressing DAT, dopamine receptor D1 (Drd1a-Cre) or dopamine receptor D2 (Drd2-Cre). The goal was to focus on glutamatergic and dopaminergic endings in the nucleus accumbens (“reward center”; [1, 2]) and the ventral tegmental area, in which DAT and either D1 or D2 receptors are expressed. Based on the role of Kalirin in responses requiring GluN receptor function and the fact that the PH1 domain of Kalirin binds to the juxtamembrane region of the C-terminal cytosolic tail of GluN2B [28] (Fig. 1a), we used ifenprodil to evaluate the role of NR2B-containing GLUN receptors.

## Results

### Cocaine locomotor sensitization reflects a lack of Kal7

Our previous studies on Kal7^KO^ mice established that the absence of Kal7 led to a marked increase in cocaine self-administration, as well as enhanced locomotor sensitization to experimenter-administered cocaine [7–9]. Since levels of Kal9 and Kal12, which play essential roles in neurite extension and branching early in development, rise in Kal7^KO^ mice [29, 30], it seemed possible that changes in these isoforms could contribute to the exaggerated responses to cocaine availability or administration. If so, the response of KalSR^KO^ mice to cocaine would be expected to differ from the response of Kal7^KO^ mice. There was a small but statistically significant increase in baseline locomotor activity in the KalSR^KO^ mice (Fig. 1c), as seen previously with Kal7^KO^ mice [7]. When KalSR^KO^ mice were tested against WT mice using the same paradigm of experimenter-administered cocaine (Fig. 1c), increased locomotor sensitization to cocaine was observed. The increased locomotion was still significant when the baseline locomotion after saline injection was subtracted (p < 0.001; not shown). The exaggerated locomotor response observed in KalSR^KO^ mice remained after 12 days of withdrawal from cocaine (not shown), as expected. Since a similar increase in locomotion was observed in the absence of Kal9 and Kal12 (KalSR^KO^) and in the presence of elevated levels of Kal9 and Kal12 (Kal7^KO^), our data suggested that the absence of Kal7 produced the increased locomotor sensitization to cocaine [7].

### Locomotor hypersensitivity to cocaine involves GluN2B

**Fig. 2:**
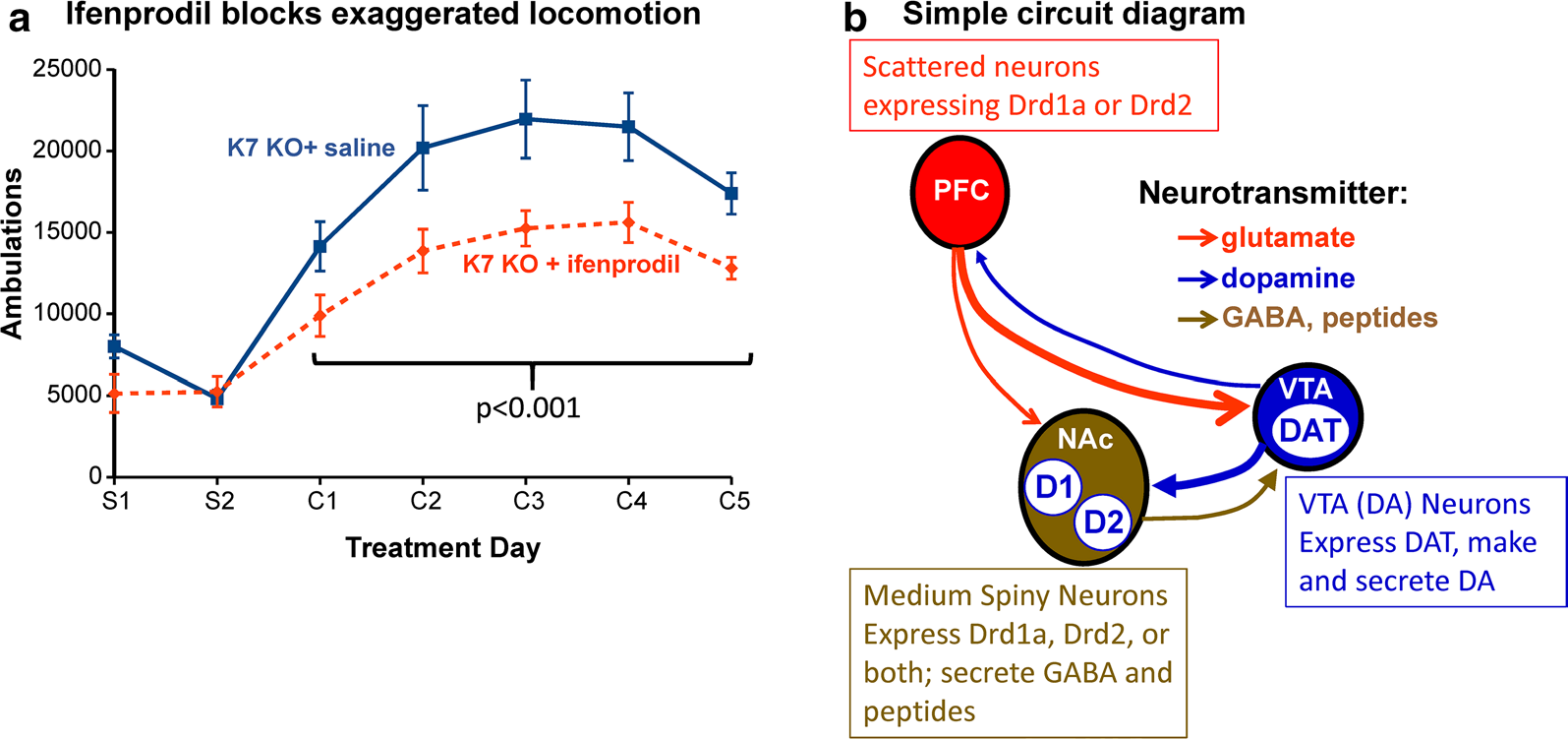
GluN2B blockade with ifenprodil inhibits cocaine locomotor hypersensitization in Kal7^KO^ mice. **a** Wildtype and Kal7^KO^ mice were given a 2 mg/kg injection (i.p.) of ifenprodil 10 min before receiving a saline or cocaine injection. Kal7^KO^ mice showed a significant decrease in cocaine-induced hyper-locomotion following treatment with ifenprodil. The cocaine response of wildtype mice was unaffected by ifenprodil (Additional file 1: Fig. S1). Males only, N = 7 each genotype. The effect of ifenprodil was barely significant for the first cocaine injection (p = 0.036) but the repeated effect of ifenprodil was highly significant (SigmaPlot using repeated measures ANOVA, p < 0.001). **b** Simple circuit diagram depicting some of the connections between the prefrontal cortex (PFC), nucleus accumbens (NAc) and the ventral tegmental area (VTA). *Darker solid lines* denote connections believed to be enhanced by prolonged cocaine exposure, while *dashed lines* depict connections thought to be weakened by cocaine [3, 4, 35–37]. Cre-recombinase mediated decrease in Kalirin expression will occur in the indicated neurons

Administration of a low dose of the GluN2B blocker ifenprodil abrogated the differences in cocaine conditioned place preference and in passive avoidance fear conditioning between WT and Kal7^KO^ mice, with no effect on locomotor activity [28]. We wanted to know whether administration of ifenprodil immediately before each dose of cocaine would blunt the locomotor hyper-sensitization observed (Fig. 2a). Prior administration of ifenprodil suppressed the hypersensitivity to cocaine normally seen in Kal7^KO^ mice (p < 0.001). When given to WT mice, the same low dose of ifenprodil had no significant effect on locomotor sensitization to administered cocaine (Additional file 1: Fig. S1). This dose of ifenprodil was previously established to be effective at antagonizing GluN2B-containing receptors with negligible effect on GluN2A-containing receptors, and no effect on baseline locomotion [31–34]. Our data indicated that the elevated locomotor response to cocaine observed in Kal7^KO^ mice was dependent on signaling that involved GluN2B-containing receptors.

Many studies have contributed to the identification of glutamatergic inputs from the medial prefrontal cortex, amygdala and hippocampus onto Drd1 and Drd2 positive GABAergic medium spiny neurons in the nucleus accumbens (Fig. 2b) [3, 4, 35–37]. Similarly, glutamatergic fibers from the hypothalamus innervate the dopaminergic neurons of the ventral tegmental area [38, 39]. Reasoning that a lack of Kal7 expression in neurons expressing DAT, the Drd1a dopamine receptor, or the Drd2 dopamine receptor would impair their ability to receive and respond appropriately to glutamatergic signals, we decided to conduct behavioral studies in the offspring of Kalirin conditional knockout mice (KalSR^CKO^ and Kal7^CKO^) paired with mice selectively expressing Cre-recombinase in these neurons. In addition, the Drd2 dopamine receptor and several elements of glutamatergic transmission have been strongly implicated in the genetic causes of schizophrenia [40]. All currently available drugs to treat schizophrenia are believed to act primarily by blocking Drd2, and to date, effective therapeutic drugs based on other target molecules have not been developed [40].

### Identification of neurons in which Kalirin expression is ablated

Given the controversies and problems with the cellular and tissue specificity of several commonly used lines of Cre recombinase mice [41–46], we first verified that our mouse lines were performing as reported. We had solid reasons to expect that the lines would be correct, given previous work on the specific DAT-Cre line we used [47] and the particular Drd1a-Cre and Drd2-Cre lines chosen [48], but a major impetus to do these tests was the unexpected occurrence with proopiomelanocortin (POMC)-Cre mice of the findings of Cre activity outside the population of cells that express POMC in the adult [43].

**Fig. 3:**
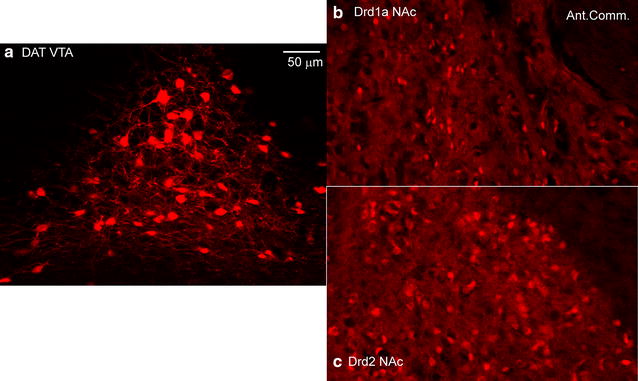
TdTomato expression in Cre recombinase mice. DAT-cre, Drd1-cre, and Drd2-cre female mice were crossed with homozygous Rosa26-TdTomato male mice. Adult progeny were perfusion fixed. The brains were sectioned and widespread regions were examined for TdTomato expression. Expression of TdTomato indicates that the DAT, Drd1a, or Drd2 promoter was active in these cells at some stage of development and identifies the cells in which expression of all Kalirin isoforms (KalSR^CKO^) or Kal7 (Kal7^CKO^) would be eliminated. **a** TdTomato expressing cells were prevalent in the ventral tegmental area in DAT-cre mice. **b** TdTomato expressing cells were prevalent in the nucleus accumbens in Drd1a-cre mice. **c** Drd2-cre mice also displayed TdTomato expression in the nucleus accumbens. *Scale bar* 50 µm for (**a**)–(**c**)

We crossed the Cre recombinase mice with the tdTomato reporter line used previously, which shows no background without Cre [43]. For the DAT-Cre mice (Fig. 3; Additional file 2: Fig. S2), tdTomato expression was detected in the tyrosine monooxygenase-producing neurons of the ventral tegmental area and substantia nigra, along with a few scattered neurons in the lateral septum. For the major dopamine-responsive neurons, the Drd1a-Cre and Drd2-Cre mice mated with the tdTomato reporter showed many neurons in the nucleus accumbens, surrounding the anterior commissure, and extending through the whole striatum (Fig. 3b, c; Additional file 3: Fig. S3, Additional file 4: Fig. S4), along with scattered neurons throughout the cortex and hippocampus. These results matched published distributions, with scattered neurons in other brain regions, as expected [47–51].

### Kalirin in DAT-expressing neurons affects locomotor hypersensitivity to cocaine

**Fig. 4:**
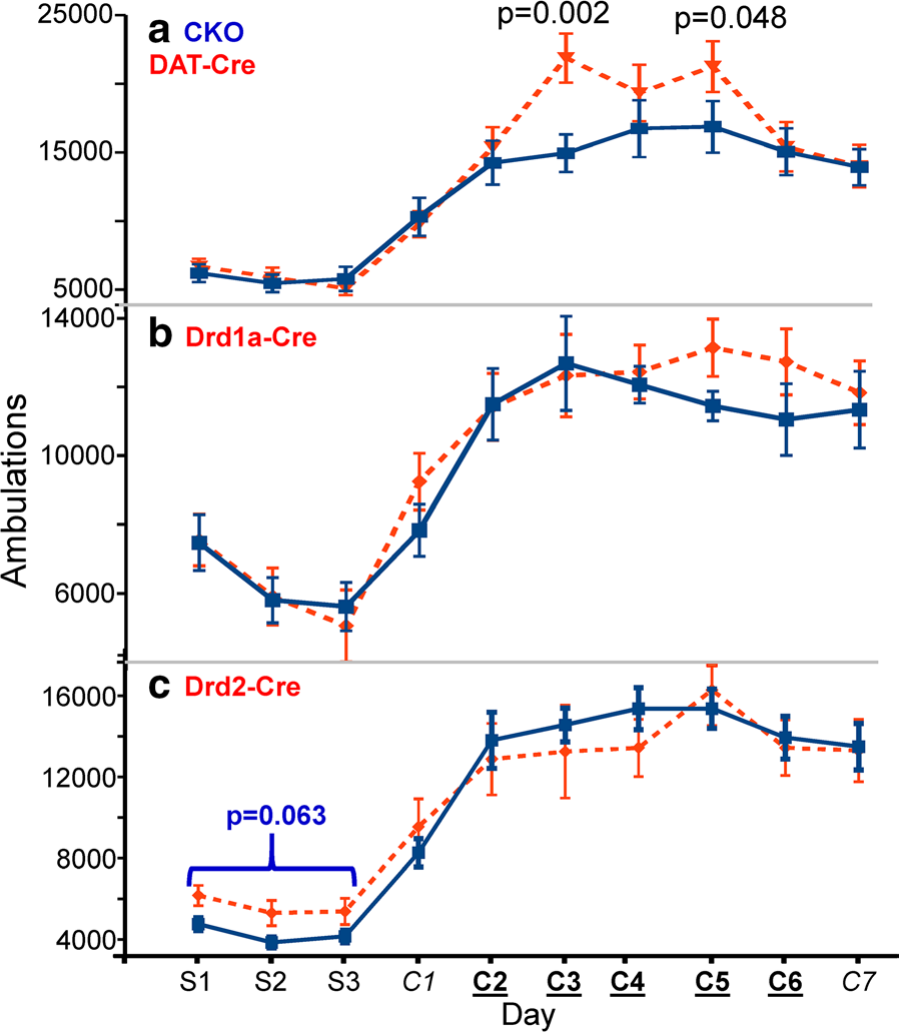
Transiently enhanced cocaine locomotor sensitization in DAT-Cre mice. **a** KalSR^DAT-KO^ and control (KalSR^CKO^) mice displayed similar locomotor responses to saline and to the first two injections of cocaine (C1, C2). KalSR^DAT-KO^ mice exhibited significantly increased ambulation on C3 (p = 0.002) and on C5 (p = 0.048) (RM-ANOVA). There were no genotypic differences on days C6 and C7 (N = 19 KalSR^CKO^, N = 20 KalSR^DAT-KO^, males only). **b** KalSR^Drd1a-KO^ and control (KalSR^CKO^) mice displayed similar locomotor responses to saline and cocaine (N = 7 KalSR^CKO^ control, N = 6 KalSR^Drd1a-KO^, males only). **c** Kal7^Drd2-KO^ and control (Kal7^CKO^) mice displayed similar locomotor responses to saline and cocaine (N = 21 Kal7^CKO^ control, N = 11 Kal7^Drd2-KO^, males only). Differences in ambulatory counts between sets of control mice were influenced by different testing venues during animal tower renovation. All testing for a single genotype was performed in one location

**Fig. 5:**
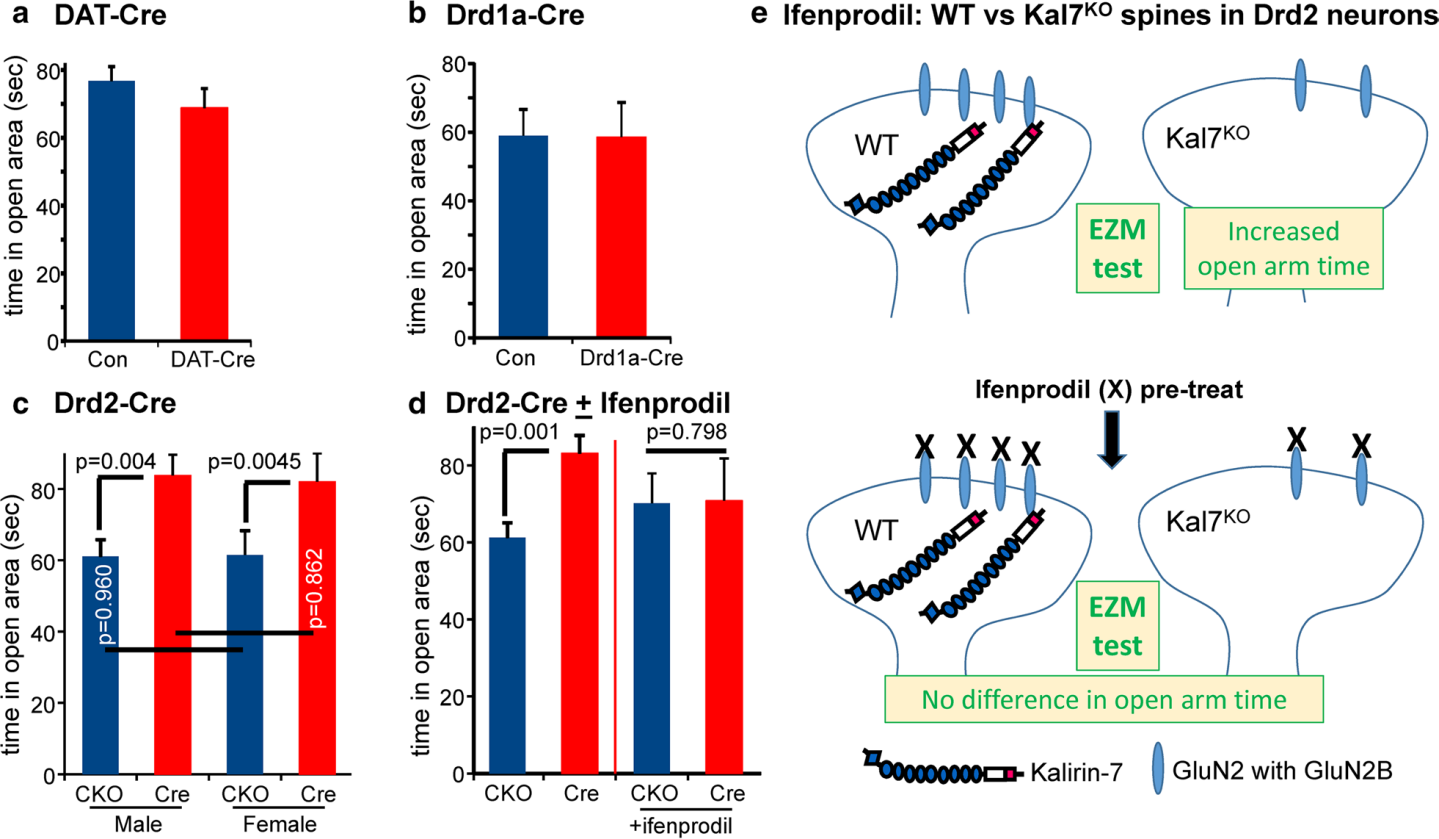
KalSR^Drd2-KO^ mice exhibited an ifenprodil-sensitive decrease in anxiety-like behavior in the elevated zero maze. **a** Male and female control (KalSR^CKO^) and KalSR^DAT-KO^ mice were tested in the elevated zero maze; open area time did not differ between controls and KalSR^DAT-KO^ mice (with no differences between male and females, data were grouped; N = 49 KalSR^CKO^; N = 54 KalSR^DAT-KO^). **b** Male and female control (KalSR^CKO^) and KalSR^Drd1a-KO^ mice were tested in the elevated zero maze; open area time did not differ between controls and KalSR^Drd1a-KO^ (with no sex differences, data were grouped; N = 7 KalSR^CKO^ control; N = 12 KalSR^Drd1a-KO^). **c** Both male and female Kal7^Drd2-KO^ mice exhibited significantly reduced anxiety-like behavior (p < 0.005) compared to control mice (Kal7^CKO^) (N = 29 Kal7^CKO^, N = 20 Kal7^Drd2-KO^ males; N = 18 Kal7^CKO^, N = 11 Kal7^Drd2-KO^ females). **d** Control (Kal7^CKO^) and Kal7^Drd2-KO^ mice were given a 2 mg/kg injection (i.p.) of ifenprodil 10 min before testing for 5 min in the elevated zero maze. Since there was no sex difference (**c**), data for males and females were pooled. Ifenprodil pretreatment abrogated the genotypic difference (N = 11 each, Kal7^CKO^ and Kal7^Drd2-KO^; pooled data from (**a**) were repeated for comparison). **e** Model of dendritic spines in Drd2-expressing neurons of WT and Kal7^KO^ mice and elevated zero maze response (EZM) before and after blockade of GluN2B-containing GluN receptors by low dose ifenprodil. Model incorporates the decrease in GluN2B seen after Kal7 knockout [9] and the direct interaction of the PH domain of Kal7 with the cytoplasmic tail of GluN2B [28]

We tested the response of mice lacking Kalirin expression only in DAT-, Drd1- or Drd2-expressing neurons to our standard experimenter-administered chronic cocaine paradigm. When Kalirin expression was eliminated in DAT-Cre-recombinase expressing neurons (KalSR^DAT-KO^), clear changes in locomotor hypersensitization were observed (Fig. 4a). The response of KalSR^DAT-KO^ mice to their initial injection of cocaine did not differ from that of control mice (Fig. 4a). Since our previous studies showed that CKO mice lacking Cre were identical to WT mice biochemically and behaviorally (total KalSR [52] and Kal7 [12]), both were often used as littermate controls, depending on the breeding strategy. Enhanced locomotor hypersensitivity to cocaine was apparent at intermediate times (C3 and C5), but this response was lost with additional injections of cocaine. For mice expressing Cre-recombinase in Drd1a- or Drd2-expressing neurons, locomotor hypersensitization to cocaine was similar to that in wild type mice (Fig. 4b, c). Interestingly, the Drd2-Cre mice showed mildly elevated locomotor activity at baseline, which correlates with the decrease in anxiety-like behavior observed in Drd2-Cre mice (Fig. 5).

### Kalirin in Drd2-expressing neurons affects anxiety-like behavior

In addition to its central role in the reward pathway and the addictive response to cocaine, the nucleus accumbens plays an important role in anxiety-like behavior. We demonstrated that global elimination of Kalirin expression or specific elimination of Kal7 (KalSR^KO^ and Kal7^KO^) produces a decrease in anxiety-like behavior; both KalSR^KO^ and Kal7^KO^ mice spend more time in the open area of the elevated zero maze than control mice [12, 52]. The KalSR^DAT-KO^, KalSR^Drd1a-KO^ and KalSR^Drd2-KO^ mice were tested in the elevated zero maze (Fig. 5a–c). There were no sex or genotype dependent differences seen in time spent in the open area in mice with Kalirin expression knocked out in DAT- or Drd1a-expressing neurons (Fig. 5a, b).

In contrast, elevated zero maze testing of both male and female mice lacking Kalirin expression only in Drd2-expressing neurons showed a decrease in anxiety-like behavior (increased exploratory behavior); male and female mice unable to express Kalirin in Drd2-expressing neurons spent more time in the open area of the maze (p < 0.005) (Fig. 5c). The magnitude of the effect was similar to that observed when Kalirin expression was eliminated in all neurons (Fig. 1c), suggesting an important contribution of Kalirin expressed in Drd2-expressing neurons in normally suppressing anxiety-like behavior.

We next used pretreatment with ifenprodil to ask whether the decrease in anxiety-like behavior observed in the KalSR^Drd2-KO^ mice reflected altered signaling at excitatory synapses that utilized GluN receptors containing the GluN2B subunit. Earlier studies comparing global Kal7^KO^ mice versus wildtype mice demonstrated that pretreatment with ifenprodil abrogated the genotypic differences in cocaine conditioned place preference and in passive avoidance, with no effect on baseline ambulations [28]; these studies confirmed the lack of effect of ifenprodil on baseline ambulations (not shown). The decrease in anxiety-like behavior observed in the KalSR^Drd2-KO^ mice was abrogated by prior administration of a low dose of ifenprodil; after ifenprodil pretreatment, KalSR^Drd2-KO^ and KalSR^CKO^ mice spent similar amounts of time in the open arms of the elevated zero maze (Fig. 5d, e). The reduced anxiety-like behavior observed in Kalirin knockout mice is crucially dependent on loss of Kalirin function in neurons expressing D2 dopamine receptors and requires normal functioning of GluN2B-containing GluN receptors in these neurons.

### Other behavioral tests

Other tests such as novel object recognition, rotarod, and grip strength [52] demonstrated no differences among the dopamine system Cre recombinase mouse lines tested (data not shown).

## Discussion

The GABAergic medium spiny neurons of the nucleus accumbens play a key role in reward pathways [3, 4, 35–37]. Chronic exposure to cocaine is known to produce multiple alterations in glutamatergic signaling [1, 5, 25, 26, 53–57], and Kal7 is localized to the post-synaptic density of glutamatergic synapses [12, 13]. In wildtype mice, expression of transcripts encoding GluN2B increases during both cocaine self-administration and chronic cocaine administration [8, 10], and earlier electrophysiological and biochemical studies revealed an essential role for Kal7 in responses that involve the GluN2B subunit [11, 28]. Medium spiny neurons expressing dopamine receptors of the Drd1 and Drd2 subtypes respond to dopamine released by fibers from the ventral tegmental area (VTA). The ability of cocaine to block dopamine reuptake by dopamine transporters (DAT) expressed by VTA neurons is essential to its profound long-term effects on behavior [1–5]. The absence of Kal7 in medium spiny neurons or in VTA neurons would be expected to affect their ability to respond to glutamatergic inputs.

These studies began with our finding of greatly increased cocaine self-administration and cocaine-induced locomotion in Kal7^KO^ mice [7–9]. Expanding these studies to KalSR^KO^ mice (Fig. 1c) and observing similar increases in the cocaine locomotor response ruled out any crucial, selective role for the larger Kalirin isoforms, Kal9 and Kal12. This allowed us to focus on ways in which the absence of Kal7 in specific cell types could produce this phenotype. Pretreatment with a low dose of the GluN2B antagonist ifenprodil blocked the enhancement of cocaine-induced locomotor activity observed to Kal7^KO^ mice without altering this response in WT mice (Fig. 2a). While an important role for glutamatergic signaling in the actions of cocaine is well documented [3, 4, 35–37], our studies revealed a specific role for GluN receptors that contain a GluN2B subunit in the increased locomotor response produced by cocaine in Kal7^KO^ mice.

In many studies of the genetic causes of schizophrenia, Drd2 and several elements of glutamatergic transmission have been strongly implicated [40]. In addition, the available drugs to treat schizophrenia act primarily by blocking D2 receptors [40]. Using well-documented Cre-recombinase mice, we investigated the effects of selective loss of Kalirin in specific subsets of cells (Fig. 3, Additional file 2: Fig. S2, Additional file 3: Fig. S3, Additional file 4: Fig. S4) [47, 48, 58]. Mice unable to express Kalirin only in neurons that express Drd1 or Drd2 responded to chronic cocaine just like WT mice; the increased locomotor sensitization to cocaine in Kalirin knockout mice was not observed. In contrast, selective loss of all Kalirin isoforms in neurons producing DAT (one major site of action for cocaine) produced an initial hypersensitivity to cocaine (Fig. 4). An obvious potential site of altered neurotransmission in the KalSR^DAT-KO^ mice is the glutamatergic endings onto dopamine-synthesizing neurons in the VTA (Fig. 2b). Studies in model systems and in Kal7^KO^ mice demonstrated a role for Kalirin in endocytosis [13, 28, 59]; loss of Kalirin expression could affect surface levels of DAT in the KalSR^DAT-KO^ mice, leaving the dopaminergic terminals in the nucleus accumbens more sensitive to DA uptake blockade in the presence of cocaine.

One of the most striking results of these studies was that selective loss of Kalirin in cells expressing the D2 dopaminergic receptor mimicked the decrease in anxiety-like behavior (increased time in the open area) seen in both Kal7^KO^ and KalSR^KO^ mice (Fig. 5) [7, 52]. Interestingly, selective loss of Kalirin isoforms in POMC-Cre mice produces a similar reduction in anxiety-like behavior [43]. The anxiety-like responses captured by the elevated zero maze are quite complex and mapping the circuits involved in the altered anxiety-like responses observed in KalSR^Drd2-KO^ and KalSR^POMC-KO^ mice may provide new insight into the pathways involved. Our previous work demonstrated GluN2B direct binding to the PH1 domain of Kalirin, and showed that ifenprodil-mediated inhibition of GluN receptors containing GluN2B could abrogate both the differences in conditioned place preference for cocaine and the differences in passive avoidance learning observed between WT and Kal7^KO^ mice [28]. Peng et al. [24] confirmed that Kal7 binds GluN2B, demonstrating a role for Kal7-GluN2B in spinal cord pain perception. Interestingly, Kalirin and GluN2B are frequently co-regulated, both increasing after cocaine exposure [10] and GluN2B decreases after Kal7 knockout [9]. Based on the ability of low-dose pretreatment with ifenprodil to block Kalirin-dependent differences in cocaine-induced locomotor activity (Fig. 2) and anxiety-like behavior (Fig. 5d, e), these behaviors fall into this group of Kalirin/GluN2B-dependent behaviors. Several other behavioral tests showed no effect of selective ablation of Kalirin in selected cell types.

Both the D2 dopamine receptor and GluN receptors with the GluN2B subunit are expressed at high levels in the nucleus accumbens [60]. The actions of ifenprodil to eliminate behavioral differences between Kal7^KO^ and WT animals (Figs. 2a, 5d, e) [24, 28] must be mediated by blocking neurotransmission at pre-existing synapses, since the effects are seen 10 min after a single injection. In fact, there is a direct interaction between D2 dopamine receptors and GluN2B receptors in the striatum which is strengthened by repeated administration of cocaine [60]. This could be the mechanism whereby the GluN2B blocker, ifenprodil, attenuates the heightened locomotor sensitivity seen in mice lacking Kal7 (Fig. 2a). The interaction of D2 receptors with the GluN2B subunit interferes with the normal interaction of CaMKII with GluN2B, decreasing CaMKII-dependent phosphorylation of GluN2B, and reducing GluN2B currents specifically in striatal neurons [60]. Disruption of Kal7 expression specifically lowers the amount of cell surface GluN2B, decreasing GluN2B-dependent currents and depressing long-term potentiation and depression [11]. Cell-specific disruption of GluN2B surface expression in D2-expressing cells (in D2-Cre/Kal7^CKO^ mice) may underlie the decreased anxiety (increased exploratory behavior) in the elevated zero maze (Fig. 5c). The genetic differences observed in the elevated zero maze are also abrogated by a low dose of ifenprodil, as was previously seen with conditioned place preference and passive avoidance learning differences between WT and Kal7^KO^ mice [28] (Fig. 5d, e).

Genetic studies and analysis of post-mortem tissue have implicated Kalirin in cardiovascular disease, stroke, intellectual disability, schizophrenia, Huntington’s Disease and Alzheimer’s Disease along with substance abuse [16, 17, 19, 20, 61–69]. The various mouse models used to identify the consequences of Kalirin deficiency recapitulated several relevant human symptoms and uncovered additional deficits in bone formation, endocrine system function, pain sensation, and neurotransmission [11, 12, 23, 24, 28, 52, 67, 68, 70–72]. The judicious use of well-characterized Cre-recombinase lines and simple behavioral tests coupled with pharmacological blockers of specific forms of glutamatergic transmission should aid in the elucidation of underlying pathways and in the identification of new therapeutic approaches.

## Conclusions

The cocaine-sensitive neuronal pathways which are most sensitive to altered Kalirin function may be the pathways most dependent on GluN2B and Drd2.

## Methods

### Mice

Experiments were performed using wild type (WT) C57BL/6J, Kal7^KO^ [12] and KalSR^KO^ [52] mice. In addition, KalSR^CKO^ mice [52] were bred to Cre recombinase-expressing mice from Jackson Laboratory (DAT-Cre: JAX 006660) and the Mutant Mouse Regional Resource Centers (Drd1a-Cre; MMRRC 017264-UCD; Drd2-Cre; MMRRC 017263-UCD). Kal7^KO^ and KalSR^CKO^ mice have been backcrossed into wildtype C57BL/6 mice from Jackson Laboratory (Bar Harbor, ME) for more than 20 generations. The Cre mice were carried/propagated as heterozygotes by crossing with C57BL/6 mice from Jackson Laboratory. The breeding was usually (Cre/+, CKO/+ × +/+, CKO/CKO) so that the offspring would have a high fraction of useable mice (Experimental mice: Cre/+, CKO/CKO vs. Control mice: +/+, CKO/+ and +/+, CKO/CKO). Since our previous work established that the Kalirin proteins and behavior are normal in the CKO animals (total KalSR [52] and Kal7 [12]), we treated all the littermate CKO animals with no Cre as control. In some experiments, the breeding strategy was not as efficient; we bred male Cre/+, CKO/+ with female +/+, CKO/+, which yields Cre/+, CKO/CKO (cell-type-specific knockouts) and fully WT mice, among others. All testing was with littermates, regardless of breeding strategy. Homozygous Rosa26-TdTomato mice (JAX# 7905; B6.129S6-Gt(ROSA)26Sortm9(CAG-tdTomato)Hze/J) were used as reporters for the sites of Cre expression, visualized as described [43]. Mice were group-housed in the University of Connecticut Health Center animal facility on a 12-h light/dark cycle (lights on, 7:00 a.m.). Food and water were available ad libitum. Experiments were conducted in accord with University of Connecticut Health Center Institutional Animal Care and Use Committee guidelines.

### Genotyping

DNA prepared from ear and/or tail snips was used for genotyping. Genotyping of KalSR^CKO^ and KalSR^KO^ mice was performed as described [52] (Fig. 1b). Similarly, genotyping protocols for Kal7^CKO^ and Kal7^KO^ mice were followed as described [12]. Screening of genomic DNA for the presence of Cre-recombinase utilized primers Cre-F (GATATCTCACGTACTGACGG) and Cre-R (CCTTAGCGCCGTAAATCAATC). PCR conditions: 94 °C, 3 min; 94 °C, 30 s; 50 °C, 1 min; 72 °C, 30 s; 37 cycles; 72 °C, 5 min. The product of the PCR was of the predicted size (280 nt) and DNA sequence analysis confirmed its identity. Genotyping from ear punches used to identify pups was initially confusing; ear punches, but not tail clips, from Drd1a-Cre promoter mice indicated that partial excision of the floxxed allele had occurred (Additional file 5: Fig. S5). The apparent explanation is that a minority of cells in the ear punches express the Drd1a receptor gene transiently during development and cell migration to form the external ear. The visible external ear is formed by neural crest-derived cells [73]. All behavioral data from Drd1a-Cre mice used tail clip identification of genotype. This partial excision in the ear was not seen in DAT-cre or Drd2-cre expressing mice.

### Behavioral testing

Physiological observations were performed on all mice, including body weight measurement over time and general observations such as obvious tremor and ability to ambulate freely in the home cage. For all behavioral studies, male and female littermates between 80 and 120 days of age were tested during the light phase.

The cocaine sensitization daily dosing paradigm used [three intraperitoneal injections of saline (S1 to S3) followed by 10–20–20–20–20–20–10 mg cocaine (C1 to C7)/kg/d] was adapted from Pierce and Kalivas [74] and Mazzone [9]. Injections were performed on top of the home cage, and mice were immediately placed into the open field apparatus. Ambulation ratios (C7/C1 and C1/S3) were used as reliable measures of sensitization [7, 9, 74, 75]; this cocaine dosing regimen does not produce noticeable stereotypy. Where noted, ifenprodil (Tocris Bioscience; dissolved in water) injections [28] were administered intraperitoneally at 2 mg/kg 10 min before the saline or cocaine injection. This dose of ifenprodil is effective at antagonizing GluN2B-containing receptors with negligible effect on GluN2A-containing receptors [31–34].

The elevated zero maze was used to assess anxiety-like behavior [12]. Elevated zero maze and passive avoidance protocols were carried out as described [12]. Ifenprodil injections were administered intraperitoneally at 2 mg/kg 10 min before placing the animal into the elevated zero maze.

Object memory was assessed using the novel object recognition test. Motor function and coordination were assessed using the rotarod; muscle strength was assessed via a grip strength meter. Protocols for the novel object recognition test, rotarod, and grip strength test were described previously [52].

### Statistical analysis

Experiments were performed over a several year time period, much of it during renovation of the animal tower, in various temporary venues, resulting in values for baseline ambulations which varied between experiments. For open field locomotion, up to 20 mice were tested as a cohort (half CKO and half CKO with the appropriate Cre recombinase; 0700-noon), so comparisons were always made within one experimental cohort. As appropriate, ANOVA and RM-ANOVA calculations were performed using SigmaPlot 11.0 or GraphPad Prism 6 or 7.

## Additional files

**Additional file 1: Figure S1.** Ifenprodil does not block cocaine-induced increase in locomotion in wildtype mice. Testing was performed as in Fig. 2, except that WT mice were tested. Males only, N = 7.

**Additional file 2: Figure S2.** Additional sections from DAT-Cre x tdTomato mice. Additional sections from DAT-cre/TdTomato mice were examined as in Fig. 3.

**Additional file 3: Figure S3.** Additional sections from Drd1a-Cre x tdTomato mice. Additional sections from Drd1a-Cre/TdTomato mice were examined as in Fig. 3. Ant.Comm., anterior commissure; Hippoc., hippocampus.

**Additional file 4: Figure S4.** Additional sections from Drd2-Cre x tdTomato mice. Additional sections from Drd2-cre/TdTomato mice were examined as in Fig. 3.

**Additional file 5: Figure S5.** Detectable Drd1a-Cre expression in the ear but not tail. **A**. Genotyping for Cre-recombinase. **B**. Partial conversion from CKO to KO genotype detected in earclips from mice F2 and M3. **C**. Re-analysis of mice F2 and M3 using tail clips demonstrate they are CKO expressing Cre-recombinase.

## Abbreviations

GEF: guanine nucleotide exchange factor
Kal7, Kal9, Kal12: Kalirin isoforms 7, 9 and 12
Kal7^KO^: Kalirin-7 knockout
KalSR^KO^: Kalirin spectrin-repeat [total Kalirin] knockout
LTP: long-term potentiation
GluN: *N*-methyl-d-aspartate receptor
PCR: polymerase chain reaction
PH: pleckstrin homology
POMC: proopiomelanocortin
VTA: ventral tegmental area
