# Supplementary figures and images for: Using Kalirin conditional knockout mice to distinguish its role in dopamine receptor mediated behaviors
Source: BMC Neurosci. 2017 May 23;18:45. doi: 10.1186/s12868-017-0363-2 (PMC5442696; doi:10.1186/s12868-017-0363-2)

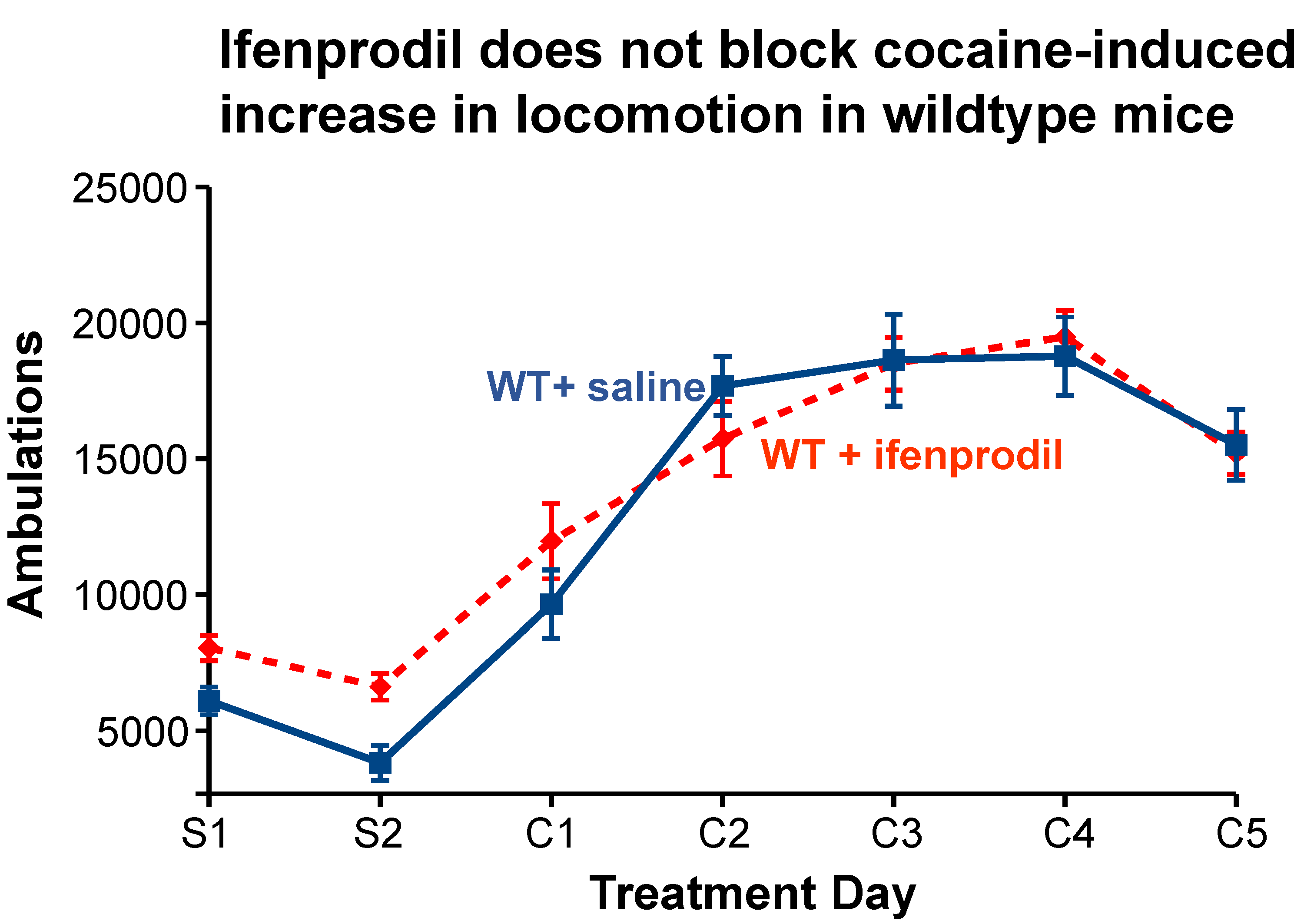

Supplement: Supplementary file 1 — Additional file 1: Figure S1. Ifenprodil does not block cocaine-induced increase in locomotion in wildtype mice. Testing was performed as in Fig. 2, except that WT mice were tested. Males only, N = 7. [file 12868_2017_363_MOESM1_ESM.tiff]

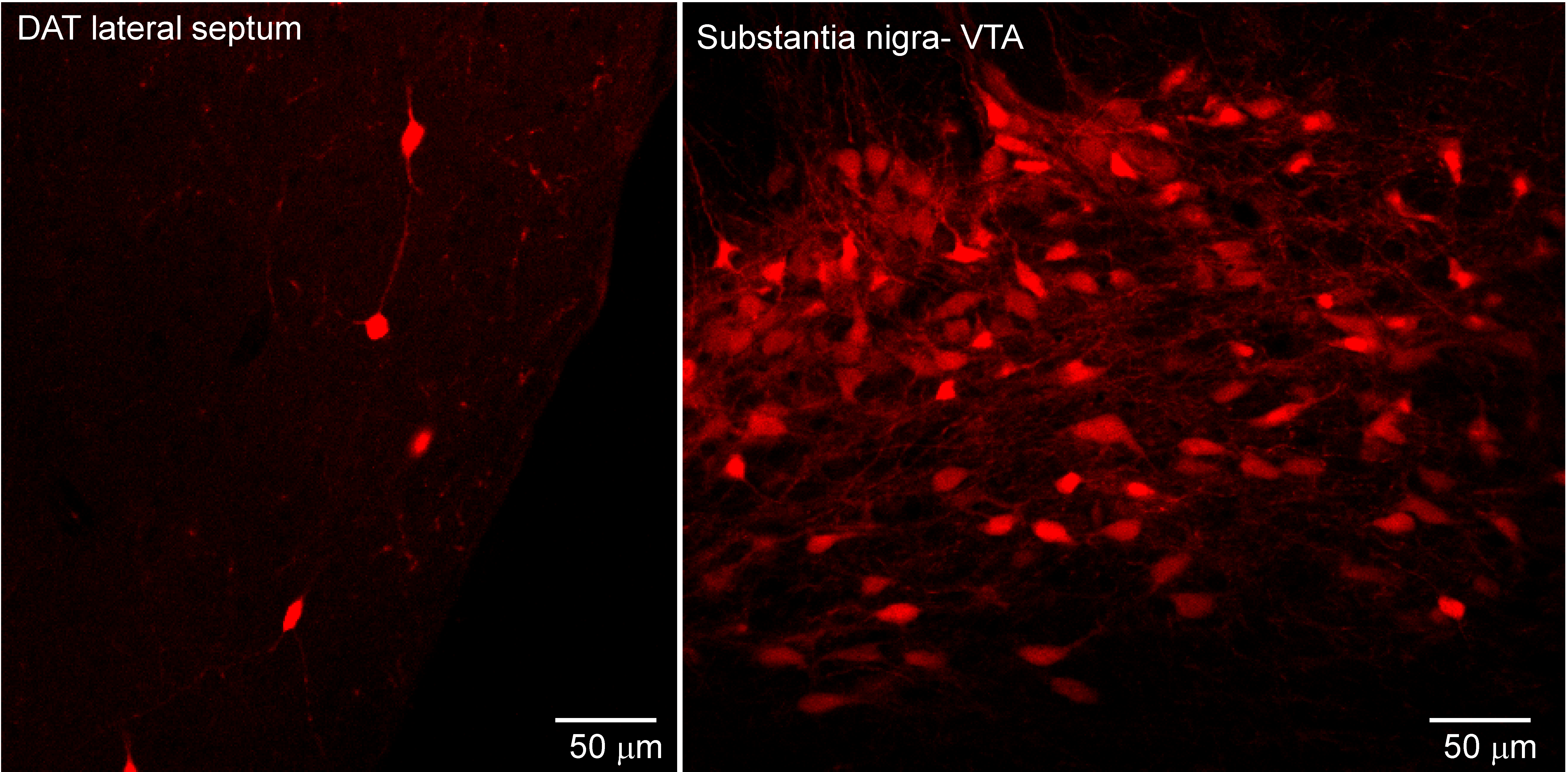

Supplement: Supplementary file 2 — Additional file 2: Figure S2. Additional sections from DAT-Cre x tdTomato mice. Additional sections from DAT-cre/TdTomato mice were examined as in Fig. 3. [file 12868_2017_363_MOESM2_ESM.tiff]

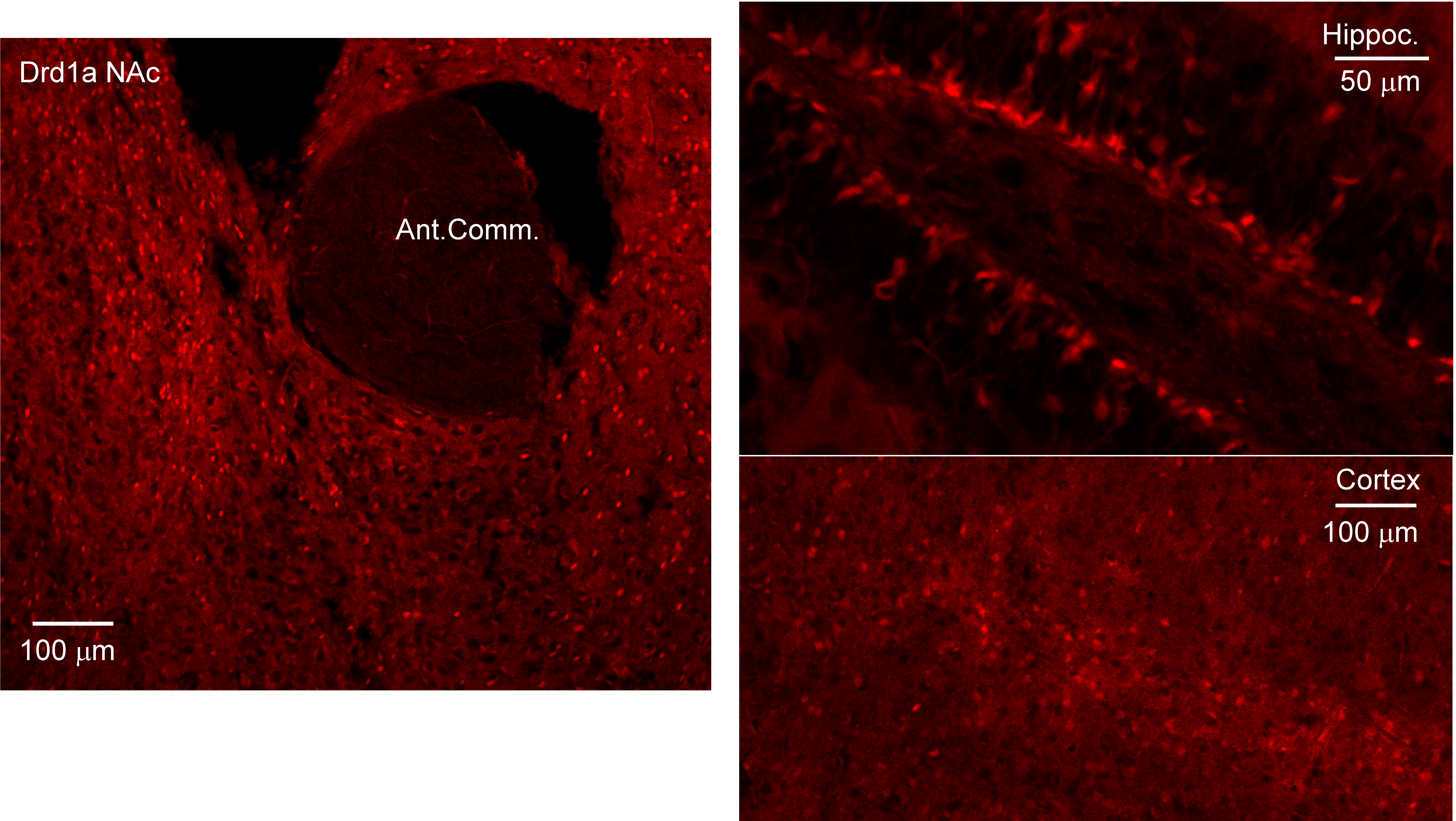

Supplement: Supplementary file 3 — Additional file 3: Figure S3. Additional sections from Drd1a-Cre x tdTomato mice. Additional sections from Drd1a-Cre/TdTomato mice were examined as in Fig. 3. Ant.Comm., anterior commissure; Hippoc., hippocampus. [file 12868_2017_363_MOESM3_ESM.tiff]

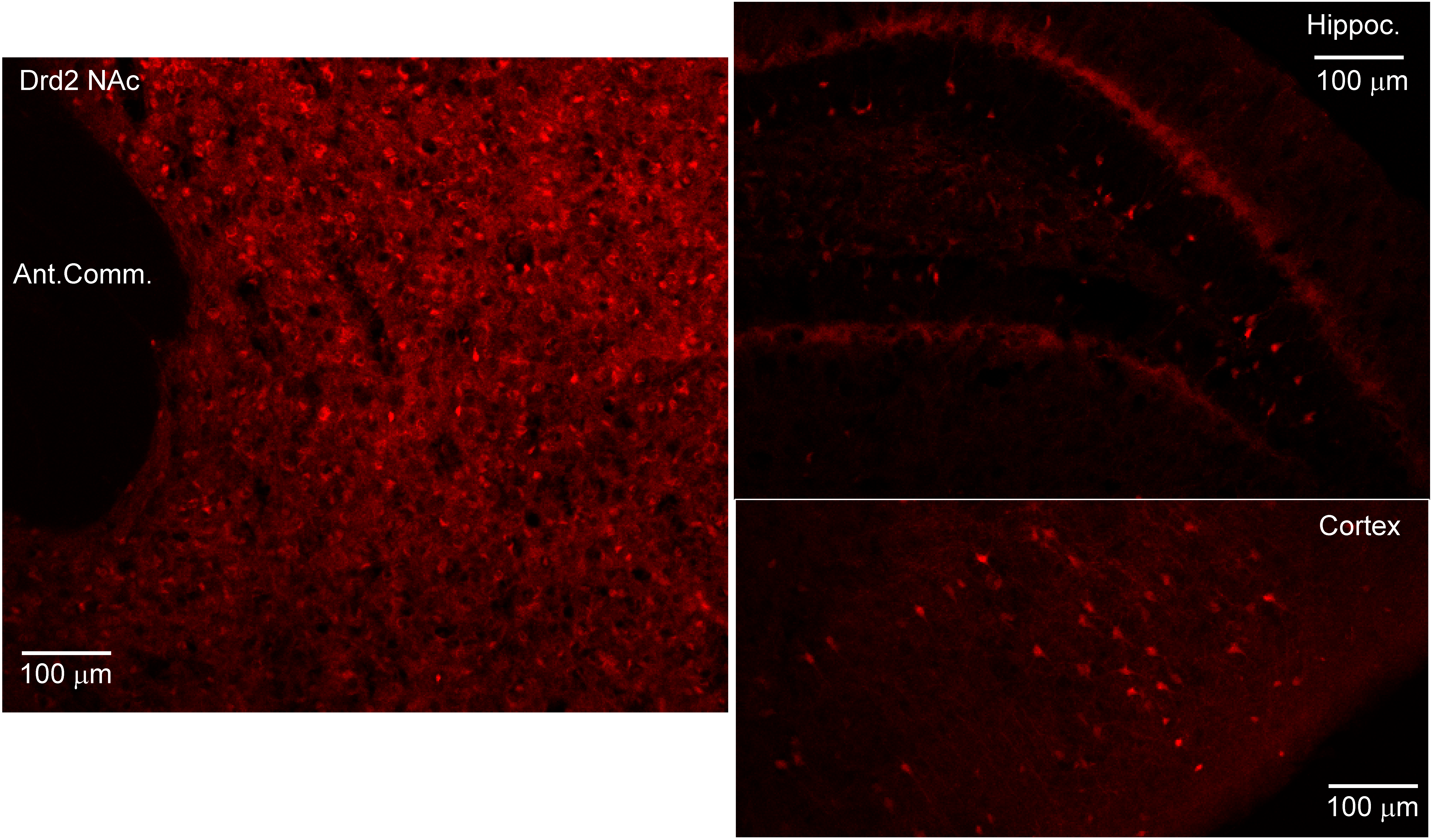

Supplement: Supplementary file 4 — Additional file 4: Figure S4. Additional sections from Drd2-Cre x tdTomato mice. Additional sections from Drd2-cre/TdTomato mice were examined as in Fig. 3. [file 12868_2017_363_MOESM4_ESM.tiff]

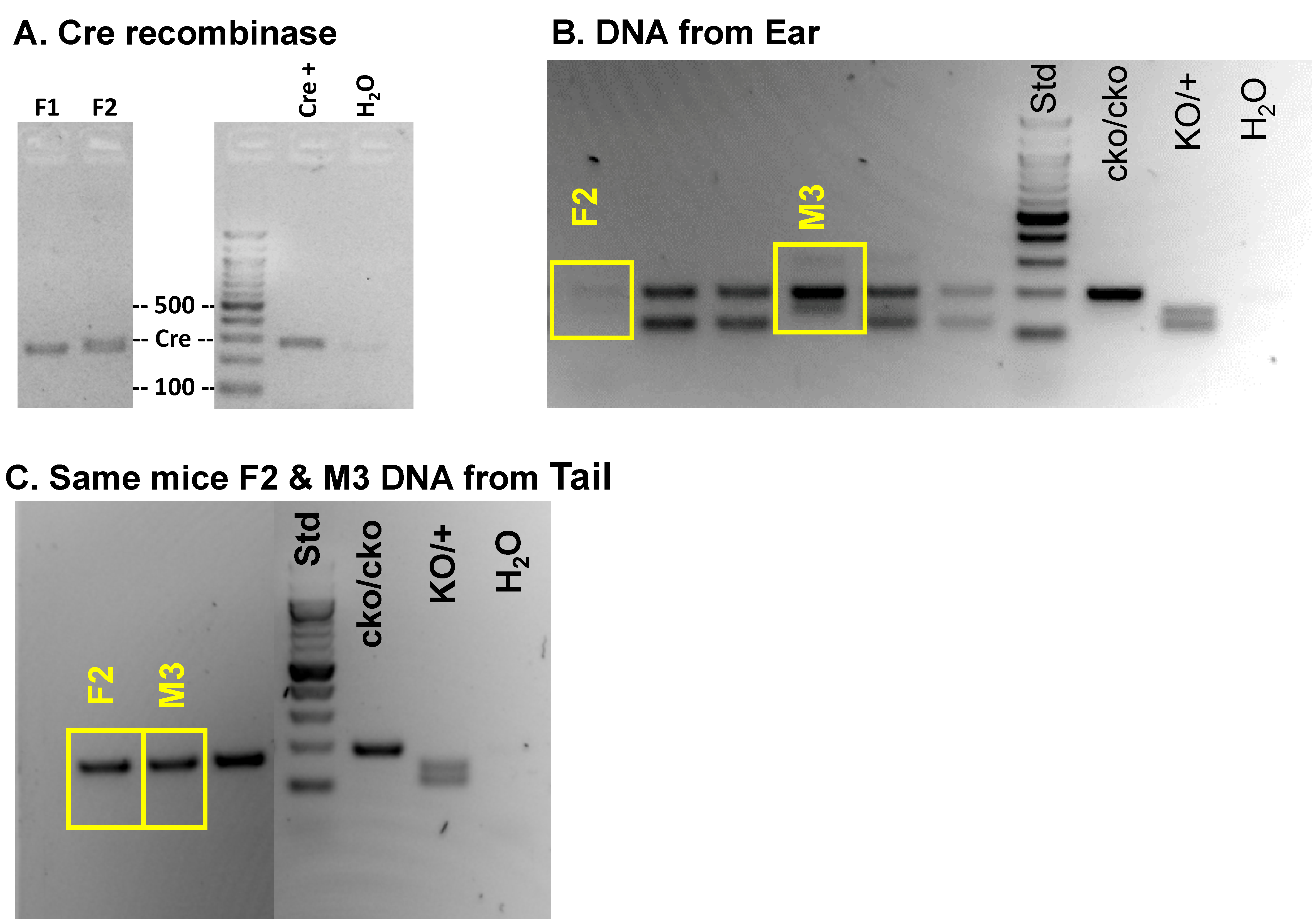

Supplement: Supplementary file 5 — Additional file 5: Figure S5. Detectable Drd1a-Cre expression in the ear but not tail. A. Genotyping for Cre-recombinase. B. Partial conversion from CKO to KO genotype detected in earclips from mice F2 and M3. C. Re-analysis of mice F2 and M3 using tail clips demonstrate they are CKO expressing Cre-recombinase. [file 12868_2017_363_MOESM5_ESM.tiff]
